# Affordability of meat for global consumers and the need to sustain investment capacity for livestock farmers

**DOI:** 10.1093/af/vfad004

**Published:** 2023-04-15

**Authors:** Peer Ederer, Isabelle Baltenweck, James N Blignaut, Celso Moretti, Shirley Tarawali

**Affiliations:** GOALSciences at Global Food and Agribusiness Network, Rapperswil, Switzerland; International Livestock Research Institute, Nairobi, Kenya; School of Public Leadership, Stellenbosch University, Stellenbosch, South Africa; South African Environmental Observation Network (SAEON), Pretoria, South Africa; Embrapa , Brasilia, Brazil; International Livestock Research Institute, Nairobi, Kenya

**Keywords:** affordability of meat products, investment capacity, livestock farming economics, protein availability, returns on livestock research, smallholder livestock farming

**Implications**

- All livestock species are in their own respective ways a key pillar of the global food system, by economic, social, and cultural values. The most frequent, but by no means the only valuable purpose, is to provide meat for food. Meat is a nearly indispensable nutrient-dense food to global consumers. While many nutrients in meat are of key importance, protein offers itself as a sentinel proxy for the analysis.
- Depending on which assumptions one makes, there is either no gap in global protein supply for human nutrition, or protein supply needs to be expanded by around 80% over current levels to meet all nutritional needs of the global citizenship. Given global demographics until 2050, the same assumptions make the difference between whether global protein supply needs to grow only by a manageable 20% or need to increase by about 150% over today.
- Independent of whether a protein (and nutrients) gap exists or not, in 2017 a minimum nutritional adequate food basket was financially out of reach for three billion people in the world, or 37% of the total population. This percentage is likely to have risen by 2022. The situation is mostly driven by the high costs for protein and other nutrients rich foods.
- The quantity and affordability gap calls for an expansion of production of all protein and nutrient-dense sources, including from animals such as meats, dairy, eggs, and fish, and at more affordable prices to the final consumer. Significant investments in livestock production systems are required, especially in lower-income countries. Investment conditions in these countries are, however, poor due to high levels of debt, weak and fragmented institutional capacity, weakly developed commercial markets, and low supply of human capital, all of which exacerbate the challenge.
- Application-oriented research and concerted strategic actions have proven to be successful tools to raise investment levels in livestock production systems and making them economically, socially, and environmentally sustainable.
- Livestock farming holds good potential for increasing food security and improved environmental performance, which also applies as much to smallholders. Smallholder’s current often poor productivity is not caused by their size, but by a lack of coordinated sectoral strategy and a lack of capital investment. When combined with innovative business models and nationally aligned policies, smallholder farming thrives on all performance dimensions, ecologically, culturally, socially, and environmentally.

## Introduction

It is estimated that global livestock production accounts for 40% of the total agricultural GDP (Salmon et al., 2020). Using the World Bank/FAOStat share of 4.3% of agricultural value added in global GDP in 2021, and IMF’s forecast of the global economy being USD 104 trillion in 2022 (current USD), this makes the global livestock sector a USD 1.8 trillion industry in 2022, of which meat comprises around two-thirds (dairy and eggs the other third) (World Bank; IMF, 2022). In terms of final consumer expenditure, animal-sourced foods including fish cost 7.0 trillion 2018 purchasing-power-parity-adjusted (PPP) USD on the global retail counter, or around 12% of the world’s total (value added of restaurants not included; World Bank, 2022; GOALSciences, 2023).

Animal husbandry, including all meat-producing animals, comprises much more than these bare GDP and consumption numbers suggest. According to the World Farmers Organisation, livestock is the most frequent kind of private capital ownership in the world, and more often for women than men. Especially in Africa and South Asia, home to 800 million bovines and thus almost half of that species global stock, animals are a financial asset providing diverse instruments for insurance and credit. They are also fundamental to the educational, cultural, and social contexts of societies. Grazing animals provide essential biodiversity and ecosystem services described elsewhere in this issue (Thompson et al., 2023). Beyond the commercial value of foods and fibers derived from these animals, they also provide important economic shadow benefits. Given its significant role in the nutritional food matrix as described elsewhere in this issue (Leroy et al., 2023), meat foods are an essential component of building and maintaining human capital for the economic function of a society. In short, meat plays a leading role in achieving a prosperous and sustainable future, both economically and environmentally, while contributing much to the livelihoods and social well-being of citizens across the globe in many dimensions.

This article examines four questions to dissect the economics of meat. First, in terms of nutrition, a key role of meat is to contribute bioavailable proteins and other essential nutrients. We will look at how much meat is available to the global citizenship, and whether these are sufficient amounts considering protein requirements (treating protein as the sentinel nutrient, in recognition that meats provide many different nutrients). Second, what does it cost those citizens to purchase this meat, also relative to other sources of key nutrients? Related to that, how affordable are meats to different sections of the global citizenship, and consequently, how many persons in the world can or cannot afford a nutritionally adequate diet? The third question asks which pathways exist to raise investment into livestock production, so that not only more can be produced (if that is desired), but also so that it becomes more affordable and is environmentally and socially sustainable. Here the well-proven tools of R&D and policy coordination are highlighted. The fourth question concerns the role of smallholder livestock farming which is the prevalent form of the production system in those regions where the essential nutrition gap is the largest and where the sociocultural dimensions are also the most prominent. Additionally, examples showcase how the smallholder system can be made more productive while preserving its multidimensional role in the socioeconomic texture of communities.

## How Much Meat is Available to the Global Consumer, and Does it Meet the Global Nutritional Requirements?

The answer to this question informs whether and how much more livestock production might be required. The answer is also very much contested, which seems in some part due to the many ways in which it can be measured, and in some part due to the emotionally charged debate around meat consumption. Rather than being able to introduce empirical clarity, the advent of modern sciences may have fanned the controversy. For instance, the German biochemist Justus van Liebig (1803–1873), an early pioneer in the nutrition sciences, promoted proteins as the “only true nutrient” and successfully commercialized the world’s first industrially produced meat replacement product (globally famous Liebig’s Extract of Meat, produced in Uruguay). Controversy followed almost instantly. Notions of a global protein deficiency have been widely debated since the 1930s and led to various public policy actions and industrial innovation in both directions, to either increase or decrease meat production, with both movements claiming science to be firmly on their side (IPES-Food, 2022).

The measurement problems are manifold. Availability is not equal to actual consumption, and the latter is difficult to estimate on an aggregated scale. Also, the availability of meat products per country per year masks uneven distribution across socioeconomic segments of society and across climatic seasons. An average seemingly sufficient availability per country per year almost certainly implies severe shortages for some segments of society during some periods of the year. Meats contain a variety of essential nutrients, some of which can also be supplied by other foods, some not, and some act complementary to each other. Furthermore, even after nearly two centuries of research on the topic, the nutritional sciences still do not agree on a narrow range of what is an optimal amount of supply for such nutrients—not even for protein—so that “sufficiency” has widely divergent meanings to arguing protagonists in the public debate.

**Figure 1.**
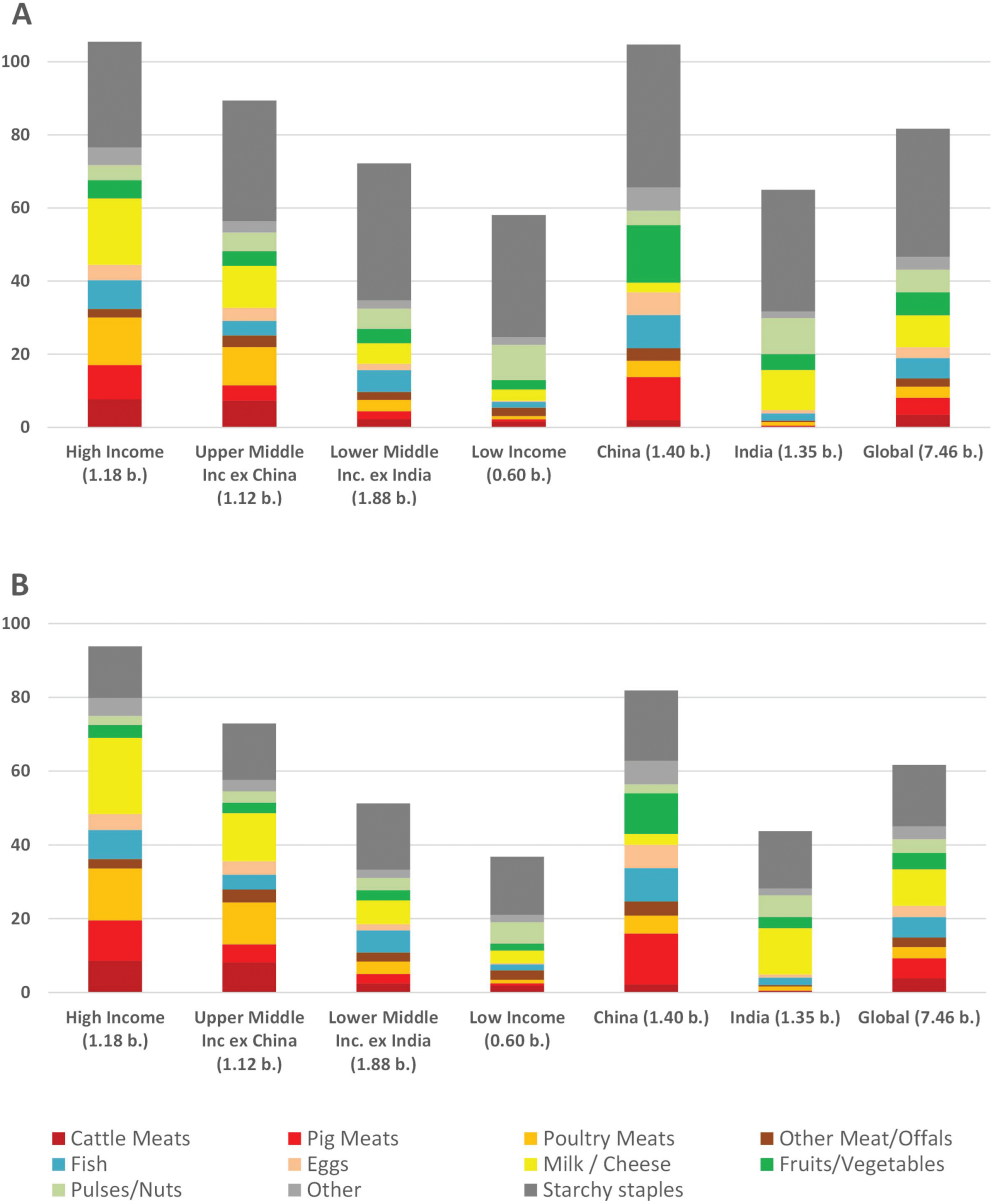
Sources of protein in gram per capita per day, average per inhabitant of country income group. Income groups are defined as per World Bank classification. Total population in billion per income group in brackets. Data source is GOALSciences (2023), calculations based on FAOStat Food Balance Sheets, reference year 2018, retrieved in January 2022. **(A)** It shows standard protein content as per FAOStat. **(B)** It shows protein content adjusted for bioavailability with DIASS scores (based on Marinangelli 2017).

This is not the place to repeat the nutritional debate, but from an economics perspective, it is relevant to establish whether there is a shortage or not, and on what main factors this would depend. The following will outline the possible answer in scenarios and is framed in the unit of protein content of foods, not because this is the only relevant nutrient, but because it provides a reasonable basis of comparison among the food groups. **Figure 1**A) describes the supply of proteins to six different income groups of countries around the world from each of the major food groups. The global average supply amounts to 82 g of protein per capita per day (pc/pd). The high-income countries and China each reach 105 g pc/pd, while low-income countries reach 58 g pc/pd. The composition of protein sources varies widely among the groups. Where in high-income countries, animal-sourced foods contribute 66% of all proteins, it is 38% in China and 17% in low-income countries.

The calculations of these values are explained in more detail at GOALSciences (GOALSciences, 2023), and are based on the FAOStat food balance sheets (https://www.fao.org/faostat/en/#data/FBS) which is the only consistently measured source of primary data on that subject that exists anywhere. One feature of the FAOStat data is that they describe availability to the consumer, and not intake. Between supply of the food and the stomach, much can happen. The food might be wasted in the retail logistics chain and not make it to the consumer. At the consumer, the food may rot in the kitchen, become leftovers on the plate, or be fed to pets. Culinary preparation with high heat or hard processing may destroy some or all the proteins and nutrients. How much of these wastes is occurring has been based on rather rough estimations based on sparse incidental analysis in the past. Only in January 2022, the much-awaited Global Dietary Database (GDD, Global Dietary Database, 2022) released a massive statistical effort to distill from more than 1500 observation studies a global and country-by-country estimate of dietary intakes. For the global average consumer, the GDD estimates total protein intake to be 64 g pc/pd. Compared to an average supply of 82 g pc/pd, this would imply a protein food waste ratio of 21%. For our scenarios, we consider waste ratios of 10%, 15%, and 20% between supply and intake (the often-quoted claim that one-third of all food is wasted (FAO, 2013), includes waste during harvesting and processing as well, which are accounted for by FAOStat in their supply figures therefore the scenarios are only 10%, 15%, 20%).

The other main constraint is the uneven supply across socio-demographic strata and climatic seasons. Different incomes, cultures, and behaviors within a country can lead to different typical diets with large variations in protein and nutrient content. In the lower-income countries, seasonal availability of foods also makes a difference. Therefore, to supply most of the population with enough protein most of the time requires an oversupply on average. Here too, the amount of necessary oversupply is essentially guesswork. We assumed 10%, 20%, or 30% for the scenarios.

The question of nutritional sufficiency has many answers. The first is to agree on what is the optimal amount of proteins in a nutritiously adequate diet. Relative to an average daily diet of 2330 kcal per person (and for our purpose disregarding all necessary nuances between males and females, the young, adult, and old, the healthy and ill, and the pregnant, lactating, or reproductive-intentioned), the US/EU recommended daily allowances suggest that 64 g pc/pd is sufficient. Researchers analyzing the least-cost healthy food baskets assumed 75 g pc/pd, or 12.8% of the caloric intake to come from proteins (see further below). Epidemiological evidence from the globally most representative and thoroughly conducted prospective PURE-study suggests 17% of the caloric intake, or 100 g pc/pd (Dehghan et al., 2017). Several popular diets such as ketogenic, Atkins, or paleo diets recommend protein shares of up to 35%. For our scenarios, we chose the two values of 75- and 100-grams pc/pd.

Finally, there is the issue of protein digestibility, for instance as expressed by the DIAAS score, whereby the source of protein is adjusted for a bioavailability index. Generally speaking, animal-sourced foods appear to have higher bioavailability to humans than plant-based sources. There is more detail on this subject elsewhere in this issue (Leroy et al., 2023). **Figure 1**B shows the protein supply adjusted by DIAAS-scores per country income group.

**Figure 2.**
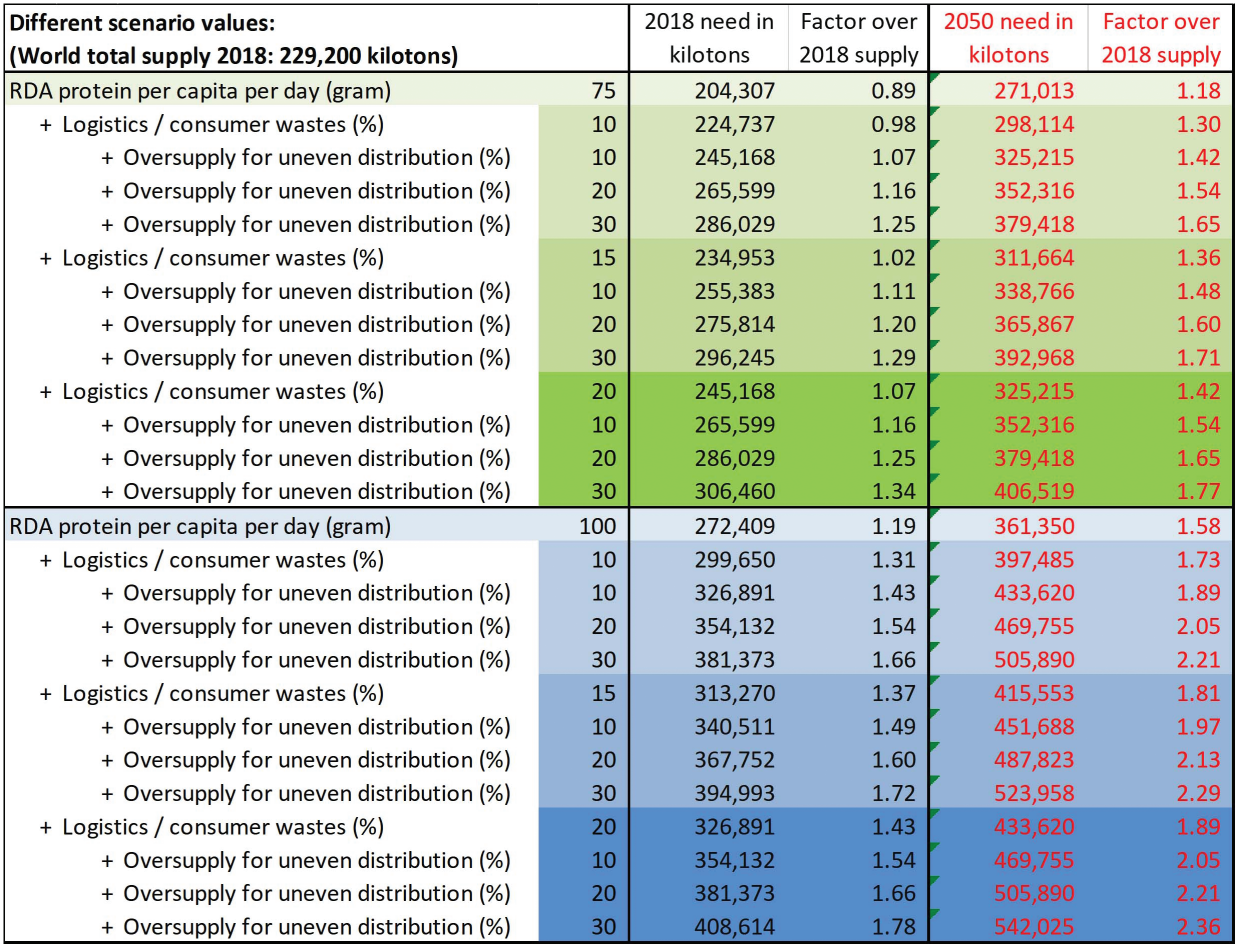
Different scenarios for estimating the global protein gap for 2018 (black numbers on the left half) and for 2050 (red numbers on the right half, then assuming global population of 9.9 billion). The table shows 40 different scenarios. Two scenario clusters are driven by whether the Recommended Daily Allowance (RDA) is either 75 g per capita per day (green, top half), or whether it is 100 g pc/pd (blue, bottom half). The next scenario cluster are assumptions on how much waste needs to be accounted for, whether 0%, 10%, 15%, or 20% (increasing darker shade). The third scenario cluster are assumptions of how much oversupply is necessary to account for uneven sociodemographic or seasonal distribution, whether 10%, 20% or, 30%. With maximum assumptions, the world would need 1.78× more protein in 2018, and 2.36× more in the year 2050 over current supply. With minimum assumptions, the world’s population is sufficiently supplied with protein in 2018, and needs only 18% more until 2050. Source: GOALSciences (2023), calculations based on FAOStat Food Balance Sheet data.

The result of the scenario analysis in **Figure 2** shows the dilemma for policy making. Depending on the assumptions, the outcomes range from there being no protein deficiency at all, towards a need to increase supply by 78%—or in numbers, out of a total of 409,000 kilotons of a currently required protein supply, only 229,000 kilotons would be available. The same assumptions then also drive the future scenarios to the year 2050. The answer could lie between a manageable necessary increase of just 18% more supply until 2050, or a need to multiply supply by almost factor 2.5× until 2050 to provide sufficient protein for everybody—depending on which assumptions one prefers, and in each case assuming that waste and demographic/climatic unevenness would not grow proportionally with additional supply.

The implications for the livestock sector of these scenarios are similarly large. On the one end of the scenarios, the examples of large protein supply with vegetables and pulses in China and India (**Figures 1** A and B) would show that on a global scale there is enough underexploited potential to expand these categories, so that the livestock industry might not need to grow at all, to meet global demand until 2050. On the other end of the scenarios, it could reasonably be argued that the global livestock sector needs to at least double its output to close the nutrient gap until 2050, even if plants and fish resources were much expanded as well. The perception of a significant gap in nutrient supply triggers high investments in alternative meat production technologies, for instance cell-based cultures or precision fermentation. However, they are unlikely to contribute much to the solution in the foreseeable future, as argued elsewhere in this issue (Wood et al., 2023).

## The Affordability of Adequate Nutrition to the Global Consumer

Various concepts exist to measure affordability. In the most recent, and so far, largest data analysis effort, a group of authors has been publishing the cost of a nutritionally adequate diet (CoNA-diet) or the cost of a healthy diet (CoHD-diet) to different income segments of a global society on a country-by-country basis (Herforth et al., 2020, 2022; Bai et al., 2022; Mahrt et al., 2022). Detailed data results based on these studies are easily accessible on the just launched interactive map display tool www.foodsystemsdashboard.org.

Those investigations relied on the uniquely and only recently created ICP dataset of the World Bank, which records price data for final consumer items for most countries around the world in 2017, including for all major food products. For the first time, this data allows a detailed and robust comparison of consumer prices for specific food items across the world. Herforth et al constructed a standardized daily food basket consisting of food group reference foods of 322 g of dry rice, 270–400 g of vegetables, 230–300 g of fruits, 210 g of eggs, 85 g of dry beans, and 34 g of oil, which would provide 2330 kilocalories, of which 12.8% (or 75 g pc/pd) are proteins, and then costed these items with the World Bank data. Country by country these reference foods would be replaced by locally common least cost items in the same food category, for instance other grains instead of rice for starchy staples, or other animal-sourced foods instead of eggs.

**Figure 3.**
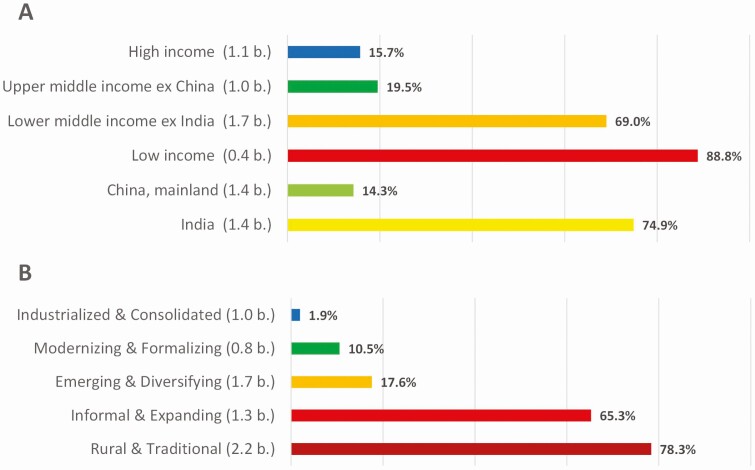
Share of population who cannot afford a healthy diet in 2017. **(A)** By country income group. Income groups are defined as per World Bank classification. **(B)** By characterization of food systems. Source for the data is www.foodsystemsdashboard.org retrieved in September 2022, which is based on Herforth et al. (2022) and Bai et al. (2022). Population is in billion in brackets; population figures do not add up to total global population because some countries are missing (in particular low-income countries).

Overall, the analysis yields that in 2017, 37% of the global population could not afford a daily healthy meal, on the assumption that food should not cost more than 52% of income. That amounts to a total of about 3 billion people, of which nearly 1 billion are from India. Three billion people is equal to the global population in the year 1960. This number masks large variation. For the 26 countries whose food systems are classified as “Rural and Traditional” the number is 78%, whereas in “Industrialized and Consolidated” food systems the number is 2% (**Figure 3**).

**Figure 4.**
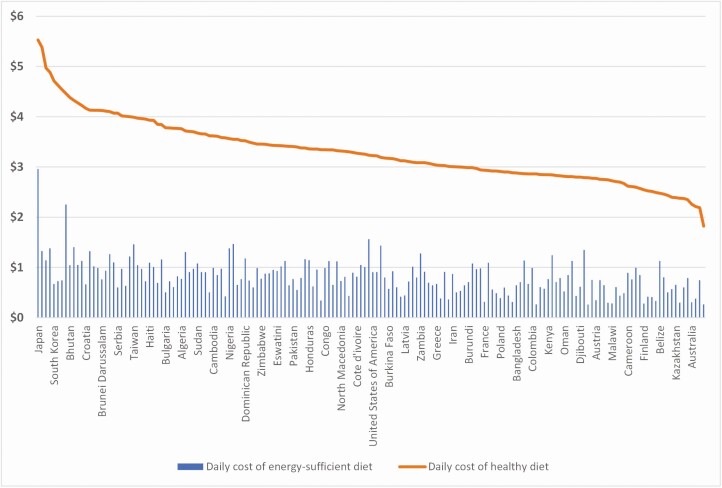
Daily cost of a merely energy-sufficient diet per person (blue bars) and a healthy diet per person (orange dots/line) per country, in PPP adjusted USD. 168 countries are shown, but only selected countries are named in the legend. Countries are arranged by cost of healthy diet, from left as the most expensive (Japan shown) to the right of least expensive (Australia shown). The daily cost of healthy diet fluctuates around 3 PPP USD in most countries, whereas the cost for a merely energy-sufficient diet is less than 1 PPP USD in most countries. Source for the data is www.foodsystemsdashboard.org retrieved in September 2022, which is based on Herforth et al. (2022) and Bai et al. (2022).

The numbers also reveal the pitfall of considering the average supply of proteins for sufficiency. Even though mainland China supplies on average 105 g of proteins pc/pd on average, there are still 14% of the Chinese population, who cannot afford a healthy basket of foods. Upper middle-income countries supply 89 g of protein pc/pd on average, but 20% of their population cannot afford nutritious meals. Across all countries, the least cost of a healthy diet is mostly around 3 PPP USD per day. A mere energy-sufficient diet costs only around 1 PPP USD per day (**Figure 4**). It is the high-quality nutrients that drive up the cost of healthy food, not the quantity of energy supplied by carbohydrate-rich foods, such as sugars and starchy staples.

**Figure 5.**
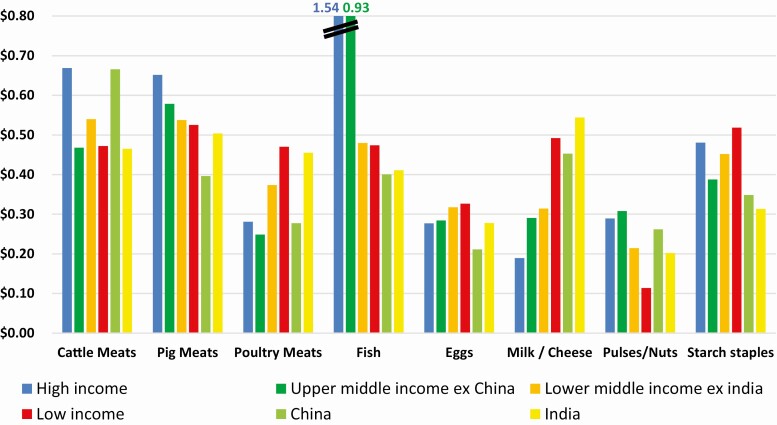
PPP (food)-adjusted retail price in USD per 10 g bioavailability-adjusted protein content in a food group item by country income groups. Income groups are defined as per World Bank classification. Protein content is derived from FAOStat Food Balance Sheets, data retrieved in January 2022. Bioavailability is adjusted with DIAAS scores (based on Marinangelli 2017, same values as in Figure 1). Retail price data and PPP for food are derived from World Bank ICP data (there only available upon application and for scientific purposes only). Calculations performed by GOALSciences (2023).

Using the same ICP data from the World Bank, we calculated the costs of food on a per-10-gram DIAAS (bioavailability)-adjusted protein content basis (for instance, we took the price of meat and applied this price to its bioavailable-adjusted protein content to make the food groups comparable with each other, since they have different protein densities and different bioavailabilities). We used World Bank’s PPP adjustment for the food category, which is different from overall country GDP PPP values. In this way, we accounted for the typical situation that food products in countries (especially lower income ones) are relatively cheaper compared to other consumption categories, and thus more affordable. (In lower-income countries the difference between GDP PPP and food PPP can be up to factor 2; therefore, our PPP USD is not directly comparable to the PPP USD of Herforth et al.) **Figure 5** shows that broadly speaking, on this basis the food groups have similar consumer prices across the different country income groups. Some items strike out. Fish is much more expensive in high-income countries compared to everywhere else in the world. Dairy products are the most affordable in high-income countries, and become significantly more expensive, the poorer the country is. The highest PPP cost for dairy is in India, which at the same time is most reliant on it for its protein supply. On a bioavailable protein basis, the starchy staples are mostly more expensive than the poultry meats, which shines a new light on the food/feed discussion and upcycling of low-quality foods to higher-quality foods through animals (as poultry feeds on these same starchy staples). At first sight, the most affordable source of bioavailable proteins are pulses and nuts. However, this view does not consider that pulses have high culinary preparation costs. Pulses contain toxic lectins and other problematic compounds and, depending on the heat source, must be cooked for a long time (up to 2 h) to become edible. Especially in lower-income countries, such cost of preparation adds considerable cost and would exacerbate the firewood problem for the environment. Pulses also require expensive storage conditions for year-round supply while meat can be harvested at any time of the year (Ngigi, 2022). **Figure 7** illustrates visually the challenge in form of physical mass flows of protein in the global food system for each of the different country income groups.

**Figure 6.**
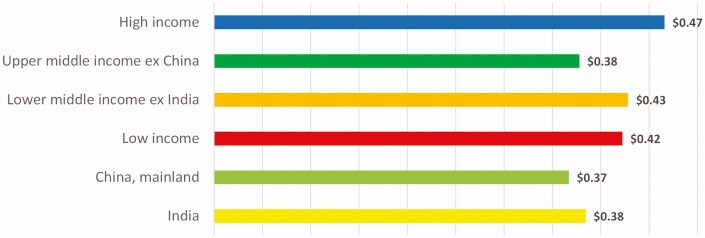
PPP (food)-adjusted retail price in USD per 10 g bioavailability-adjusted protein content of the actually available food by country income groups. Income groups are defined as per World Bank classification. Protein content of food available is derived from FAOStat Food Balance Sheets, data retrieved in January 2022. Bioavailability is adjusted with DIAAS scores (based on Marinangelli (2017) same values as in Figure 1). Retail price data and PPP for food are derived from World Bank ICP data (there only available upon application and for scientific purposes only). Calculations performed by GOALSciences (2023).

**Figure 7.**
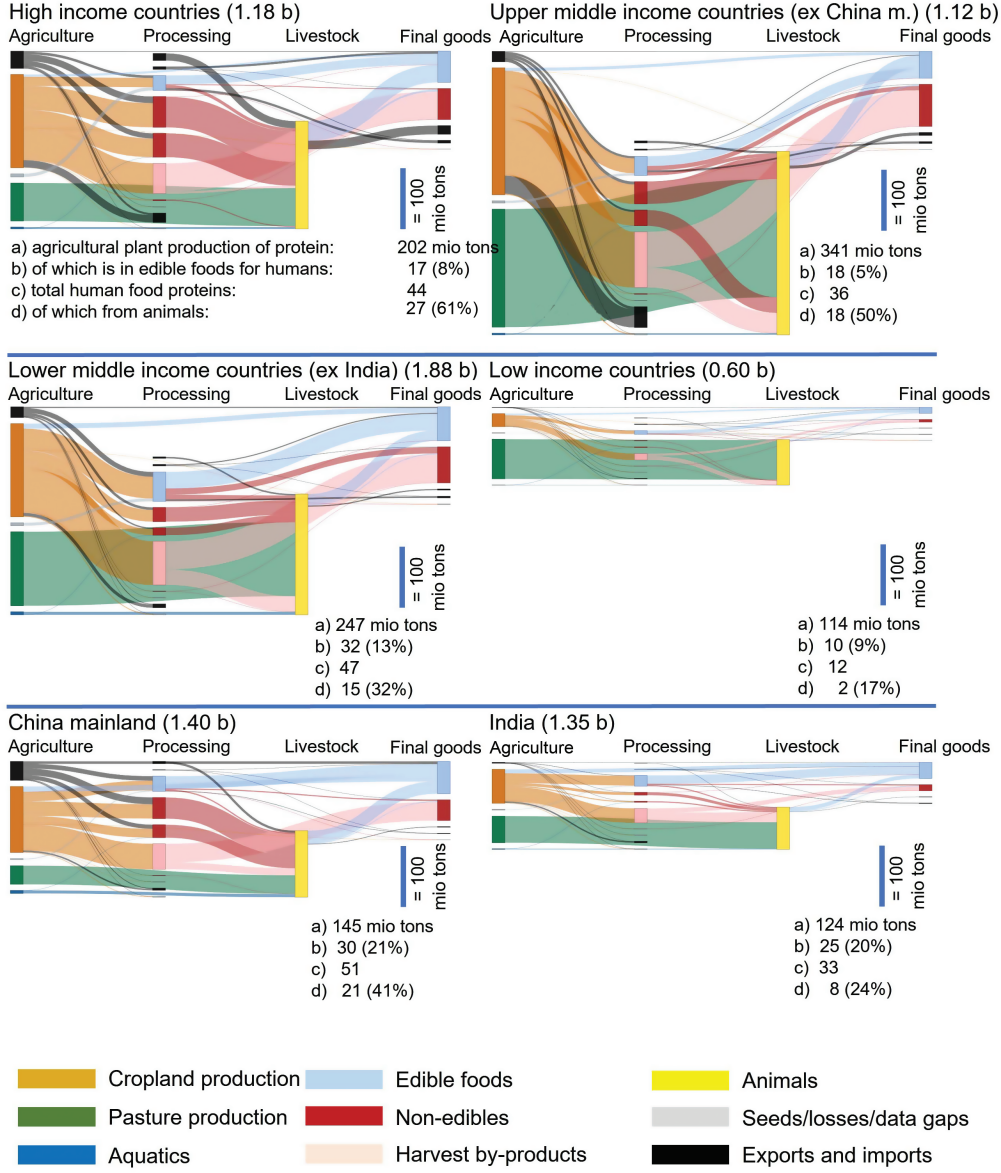
Mass flow balances of protein content from agricultural production via processing and animals towards food supply. Each flow in proportional size to each other. Excerpts from PLANET food system explorer of GOALSciences: https://goalsciences.org/planet-food-system-explorer Calculations for the year 2019.

While **Figure 5** shows the cost of each protein, **Figure 6** shows the cost composition of the basket of what consumers actually buy (i.e., what they buy is represented by the values of **Figure 1**B). Multiplying these out, yields that consumers around the world pay roughly the same for their bioavailable protein, namely around PPP (food) USD 0.40/10 g of protein. The significant difference between countries is not their cost of nutrition, but that poorer consumers cannot afford to buy enough of them.

Overall, the conclusion from these analyses is that whether there exists a global protein and nutrient gap or not, is a question of the assumptions about nutritional sufficiency one prefers to make. What is fairly certain, however, is that already in 2017, around one-third of the global population could not afford to buy a sufficiently nutritious meal. With the covid-19 pandemic, the Ukraine crisis and strong inflationary pressures arising in 2022, this percentage is likely to have increased by 2022. As the global population is set to grow from 8 billion in 2022 to 10 billion in 2050, and the increase occurring primarily in the lower-income segments, the affordability gap is likely to grow further. While that may sound like an urgent call to reduce the cost of bioavailable proteins and other essential nutrients, and possibly also to increase the amount of such protein and nutrients availability, the challenge is driven by multiple layers of interlocking dimensions, and not easily resolved. Certainly, the challenge has not gone unnoticed. The massive 2022 joint research report by FAO, IFAD, UNICEF, WFP, and WHO on the topic is called: “The State of Food Security and Nutrition in the World, Repurposing Food and Agricultural Policies to Make Healthy Diets More Affordable.” (FAO, 2022). A key outcome of the report is that many regions in the world need more animal-sourced foods.

## The Economics of Farming in General, and Livestock Farming in Particular

When the poor cannot afford to nourish themselves, then they must degrade the quality of their food intake to the point of harming their health. Lower health status erodes their economic potential, preventing them from increasing their income. The vicious cycle extends to the whole society struck by poverty. Lower-income countries or impoverished communities within higher-income countries cannot take advantage of their lower factor costs of land and labor because their agricultural systems typically have low productivity. The underlying reason is lack of capital investment in all aspects of agriculture, including livestock management, because ultimately there is not enough human capital.

How can this vicious cycle be broken? Studying the average balance sheet and profitability statement of an Australian livestock farm illustrates a key part of the challenge (**Table 1**). Australia is a good example, because the Australian livestock industry is globally recognized for being among the least distorted by national subsidies, and at the same time achieving the highest degree of export competitiveness compared to other countries.

**Table 1.** Average financial performance of broadacre farms in Australia, data for three seasonal years. Source: https://www.agriculture.gov.au/abares/research-topics/surveys/farm-performance, retrieved in September 2022. All values in Australian Dollar.

Averages per farm	2019–2020	2020–2021p	2021–2022y
*Sample of 1412 broadacre farms*			
Total cash receipts	$594,370	$699,800	$842,000
− Total cash costs	$437,180	$491,900	$565,000
= Farm cash income	$157,190	$207,800	$278,000
+ Change in trading stocks	−$31,780	$25,500	$39,000
− Depreciation & family labor	$132,580	$132,700	$134,000
= Farm business profit	−$7,170	$100,600	$183,000
+ Finance costs	$40,990	$40,990	$42,000
= Profit at full equity	$33,820	$141,500	$225,000
÷ Total farm capital value	$6,789,180	$7,336,200	$7,788,000
= Rate of return	0.5%	1.9%	2.9%
*Sample of 868 sheep and beef farms*			
Total cash receipts	$434,680	$477,000	$533,000
− Total cash costs	$335,540	$346,400	$370,000
= Farm cash income	$99,140	$130,600	$163,000
+ Change in trading stocks	−$46,920	$14,000	$36,000
− Depreciation & family labor	$107,840	$107,300	$108,000
= Farm business profit	−$55,520	$37,300	$91,000
+ Finance costs	$25,830	$25,100	$26,000
= Profit at full equity	-$29,790	$62,400	$117,000
÷ Total farm capital value	$6,023,740	$6,470,300	$6,768,000
= Rate of return	−0.5%	1.0%	1.7%

Even in what is considered to be a top-ranked performing livestock industry in the world, and in what is considered to be a good year for Australian livestock farmers, the investment rate of return on capital, is merely 2% in 2021. In 2019, it was even negative, the long-term average hovers around 1%. These numbers are not an Australian peculiarity. The core economics are more or less the same across the world, even if local circumstances of subsidy schemes, market situations and labor resources create lots of variation. This begs the question, of why any entrepreneur or investor should be spending their capital, time, and effort on what seems to be an inherently and structurally unsound business proposal, such as livestock farming. Why do farmers do this to themselves, if they could take the capital and invest in practically anything else which will yield better returns? The question points to the fact that livestock farming is not only about immediate and short-term measurable economic returns.

Agricultural economics is peculiar, and this is not the place to highlight all its particular dynamics. In short, since farmers are commodity producers with no pricing power and de facto unlimited competition, their average achievable market price will always be competed down to variable cost, leaving little to no return to capital. At the same time, any structural financial advantage ends up in an increased value of the land as the primary capital component. As a result of this dynamic, the often-stated request of “more income to the farmers” is too simplistic and will not achieve much. Structural increases in prices obtained by farmers make their land more expensive and thus depress real returns to capital back down. However, farmers are of course also not short-sighted. If that 1% rate of return was all there is to their efforts, then they would not be in the business of producing food.

For the global farmer, regardless of size and structure, to produce food in light of those low investment returns, they need an agreement with society that makes farming worth their while through other benefits. Such an agreement is informally operative in practically all high-productivity jurisdictions. The core part of the agreement is that the farmers must have ultra-secure title to the land on which they are operating. They own this land for themselves and for all future generations, and only under the most limited circumstances will this land or its utilization rights be taken from them. This secure title provides the farmer with another route of capital return, namely land value appreciation. As society becomes wealthier, and as the land produces more food thanks to increased amounts of investment, the land becomes more valuable. Where and when such an agreement has not been operating, for instance in the fiefdom-based systems of the European Middle Ages, or under communist regimes until the 1980s, or across much of today’s Africa, then food production is suppressed, because investments remain too low.

When a high-productivity farmer owns a lot of land under such a societal agreement, then land value appreciation provides much leverage for wealth creation. It is a special kind of financial return because it cannot easily be liquidated. If the land is sold and the wealth is realized into cash, then not only is the land gone for good, but the farmer also loses both the source of their working income as well as the lifestyle that goes with it. It may not be everybody’s idea of a good agreement, and it does not need to be. If there are enough farmers who find this an attractive proposal, then society has enough food.

The societal land “agreement” may then be reinforced with additional societal measures, such as explicit or implicit guarantees that there will be off-take markets that will under all circumstances buy everything which the farmer produces. The nature of these off-take guarantees can vary a lot, ranging from purely private cooperative arrangements to explicit state guarantees, but in one way or the other, the institutional framework of a society is always somehow directly or indirectly involved. Food production is so important to society that the sector usually operates de facto as a public utility. As a consequence, the farming sector (at least in those jurisdictions where productivity has priority) usually receives an overproportional amount of political attention, and typically receives a basket of various societal privileges (preferential tax treatments, subsidies, special legal rights, high reputational standing, etc.), which is sometimes welcomed and sometimes maligned, but which is always due to the elementary significance of food production, and the irreplaceable need to have enough farmers being willing to produce such food in light of structurally low investment returns.

In livestock farming, all the above forces are exacerbated. A livestock farmer has much capital tied up in a living animal that could easily die or get stolen, and which needs constant daily attention, all of which gives this business an even riskier profile than typical plant farming on land. Livestock farmers, therefore need even stronger societal or communal guarantees of full ownership rights to their animals (and the lands that support the animals, where land is needed) and guarantees for off-take markets that are usually covering their costs. Otherwise, the farmer will not invest, the animal will not exist, animal-sourced foods will not be available, and neither will be the numerous other ecological and socio-cultural benefits of livestock farming.

Societies or communities which for whatever reason are not willing or not capable of providing these guarantees for land or animals, will therefore have fewer farmers producing enough surplus food, and therefore less of such food is available to society relative to the purchasing power of the population. This dynamic is reinforced the more agronomically demanding the food is, which means the more investment it requires, and therefore applies especially to all the high-value protein kinds of food, including and in particular, livestock farming.

**Figure 8.**
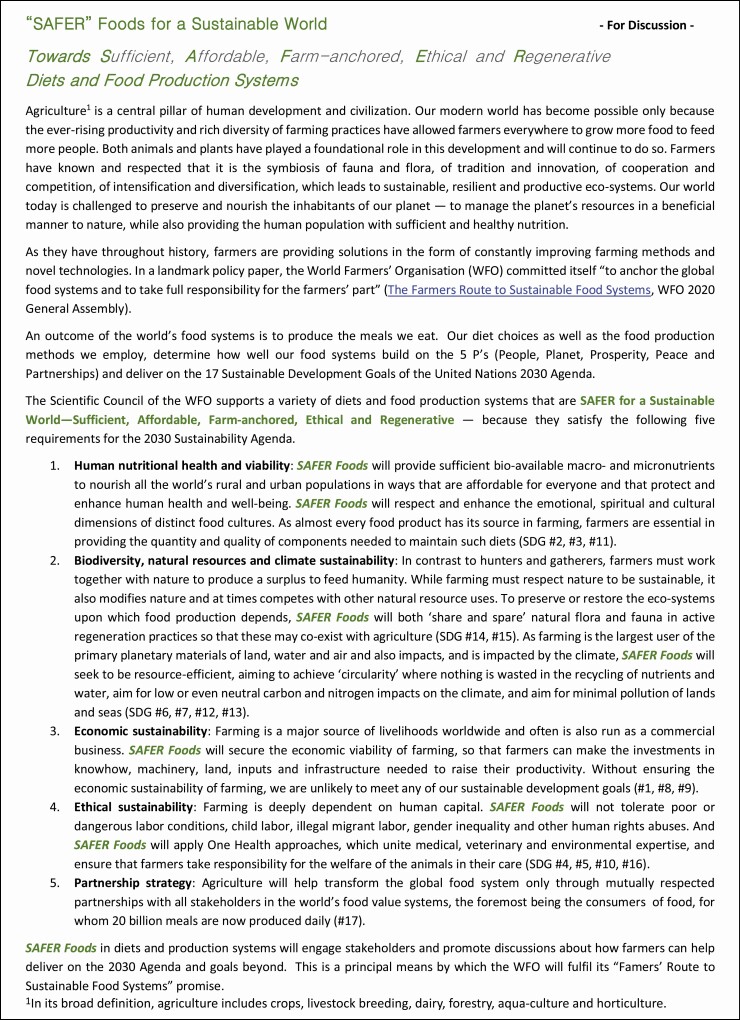
Scientific Council of World Farmers Organisation, SAFER Foods concept launched for discussion in 2020. Source: https://www.wfo-oma.org/wp-content/uploads/2021/02/WFO_SAFER-Foods-for-a-Sustainable-World.pdf

To highlight the necessity of economic sustainability of farming to be on the same standing as environmental and social sustainability for food production, the Scientific Council of the World Farmers Organisation developed an evaluation framework of SAFER Foods—towards Sufficient, Affordable, Farm-anchored, Ethical and Regenerative Diets and Food Production Systems (**Figure 8**), (World Farmers Organisation, 2020).

## How to Raise the Investment Capacity of Global Livestock Farmers, Especially in Lower-income Countries

In contrast to plant-based nutrient-rich food production from pulses, vegetables, or fruits, all of which are agronomically demanding crops, livestock has the advantage that with its wide variety of different species and production systems, it can be more easily adapted into utilizing almost any eco-system, and it can better deal with climate volatility within these eco-systems. At its extremes, camels made civilization possible in hot and dry deserts, and reindeer enabled cultures far north of the Arctic circle where plant-based agriculture is not feasible. It is this ecological flexibility that plant-based protein producers do not have, which is one reason that livestock farming should be an attractive proposition to public food security policy.

The global agriculture and health authorities, such as FAO, OIE, UNWFP, WHO, IFAD, and the global research organization CGIAR are much in alignment with the need to build on livestock production systems in lower-income countries, and to do so in line with environmental and social sustainability requirements. Multiple initiatives have been launched in recent years to facilitate scientific evidence of various kinds as a guidance to the necessary policy initiatives. One of them is the ILRI-hosted website and related projects by the Global Livestock Advocacy for Development (GLAD): https://whylivestockmatter.org/. Among other things, the website features a newly launched 2022 series of 7 briefs on livestock pathways to 2030: https://whylivestockmatter.org/livestock-pathways-2030-one-health. Another initiative is the highly active Global Agenda for Sustainable Livestock (GASL) which brings together key actors from private industry, policy, science, and international institutions to create solution spaces: http://www.livestockdialogue.org/. Another related global community is also Livestock Data for Decision (LD4D): https://www.livestockdata.org/ld4d-members. In recognition of the importance of livestock, the FAO Committee on Agriculture instituted a new subcommittee on livestock which began operation in 2022.

The highest levels of global policy communiques on how to develop the livestock sector have been issued on an almost yearly basis. For instance, in 2012 the global institutions of AU-IBAR, ASEAN, BMGF, FAO, IFAD, ILRI, World Bank, and OIE announced “A new global alliance for a safer, fairer and more sustainable livestock sector” (AU-IBAR, 2012), in 2016 the UN Committee on World Food Security issued a UN recommendation of the role of livestock on food and nutritional security: “Sustainable Agricultural Development for Food Security and Nutrition, Including the Role of Livestock” (CFS, 2016), in 2018 the Global Forum for Food and Agriculture Communique of the world’s agricultural ministers published “Shaping the Future of Livestock—sustainably, responsibly, efficiently” (GFFA, 2018), and in 2019 ILRI and WEF jointly created: “Options for the livestock sector in developing and emerging economies to 2030 and beyond. Meat: The Future Series” (World Economic Forum, 2019). In the 2021 United Nations Food Systems Summit, the role of the solution cluster “Sustainable Livestock” was particularly recognized, and the subject received substantial engagement from stakeholders.

The common theme throughout all these initiatives is twofold: 1) research and 2) concerted strategic action. Sustained and problem-solving oriented research in livestock breeds and production practices has repeatedly shown to have very high societal rates of return. For instance, 45 years of research effort by ILRI, the International Livestock Research Institute, cohosted by the Governments of Kenya and Ethiopia focusing on livestock in the Global South and being part of the CGIAR research network, has been described in the year 2020 (McIntire et al., 2020). ILRI scientific achievements can be categorized into three parts, namely animal genetics, production, and health; primary production; and livestock systems that include policy and economics, climate change research, and gender, with numerous scientific and development outcomes.

Another example of research-led success is Brazilian Embrapa, a government-owned and independently led research company to develop agriculture across the many biomes of Brazil. It was established in 1973 with the task to provide Brazil with food security and a leading position in the international market for food, fiber, and energy. To meet such a continuous challenge, in permanent dialogue with farmers, scientific organizations, and both government and civic leaders, Embrapa has been guided by the following tenets: 1) scientific excellence in agricultural research, 2) crops and livestock production efficiency and quality, 3) environmental sustainability, 4) social aspects, and 5) partnerships with the production sector (Embrapa, 2022). In 2022, Embrapa employed around 2000 researchers (221 in cattle-related) in 43 research centers working on 1036 ongoing projects. Embrapa’s research is widely credited to have created the foundation for Brazil’s agricultural success. The company calculates that for every Brazilian Real invested in Embrapa research, the society receives 23.38 Reais of economic return (1 BRL = 0.19 USD in 2022).

These experiences were also echoed by the Science Group at the UN Food System Summit, where Braun et al. highlighted that the three most immediate steps should be to increase scientific research funding, scientific research capacity, and a strengthened mechanism for science/policy interface (Braun et al., 2021).

Research and development will bear fruit if the societal conditions are strategically aligned towards promoting livestock production systems. An instrument that has proven to be particularly purposeful are so-called Livestock Master Plans (LMPs). In recognition of the under-investment in livestock, livestock master plans are mechanisms for governments to identify, verbalize, and prioritize livestock interventions. Through rigorous data collection, modeling, and stakeholders’ engagement, an LMP document provides essential evidence for enhanced and targeted investments towards sustainable livestock, both from private and public sector actors. Upfront, an LMP process recognizes the diversity of livestock species and production systems and establishes priorities which of them are particularly suited to the context of the country. Starting with the analysis of the current situation, stakeholders agree on long-term objectives to set long-term strategies and action plans. Roadmaps with specific visions, targets, challenges, strategies, and proposed investments in technology and policy interventions, with expected outputs, outcomes, and impacts are mapped out. Moreover, the process supports the capacity building of key stakeholders, for continuous update of the model based on new data and change in scenarios. The methodology has been successfully applied in Ethiopia, Rwanda, Uzbekistan, Tanzania, Bihar (India), The Republic of Gambia, and Odisha (India), with more LMPs in preparation (ILRI, 2022).

## Selected Examples of How Livestock Improves Socioeconomic Performance of Households in Lower- and middle-income Countries. Source: ILRI

- Zambia: livestock transfers increased asset accumulation by 125% and increased household incomes by 59% over 42 months
- Rwanda: one cow per poor family initiative led to 14% more assets and 8% more types of assets
- Across 6 low-income countries: livestock introduction led to 9% more assets, 14% more savings and 5% more income
- Niger: 60% of households rely on sales of animals to cope with food shortages or unexpexted medical expenditures
- Kenya: pastoralist households covered by an index-based livestock insurance were 36% less likely to be forced into distress sales of stock, 25% less likely to have to reduce the size of their meals, 33% less dependent on food aid
- Ethiopia: doubled incomes with cattle fattening programs
- Nepal: doubled incomes with goat value chain improvements
- Bangladesh: poultry interventions increased household incomes by 49%
- Kenya: a rural chicken vaccination program against Newcastle led to children having 24% more protein-rich food, which led to 1.16% more height, and 0.54% more weight

## Prospects and Promise of Smallholder Livestock Farming Systems

Nearly 50% of the world’s livestock and cereals are grown on farms of less than 20 ha. In emerging and developing economies, this number is 70% (World Economic Forum, 2019). Of those farms where livestock is a component in the farming mix (which could be at least half a billion globally), the socioeconomic livelihoods are much improved among many dimensions: in terms of absolute levels of income, in terms of income stability, and in terms of cash-surplus generation to participate in the formal economy (Staal et al., 2020). All of this contributes overproportionally to the levels of food security and educational attainment of all members of the farming household and provides important resilience against environmental and socioeconomic volatility. In most communities, it is the women who are the owners of the animals, thus serving as a gateway to gender equality.

The manifold ways in which livestock are deeply interwoven with the socioeconomic, nutritional, cultural, and health contexts of households in low- and middle-income countries are often overlooked. This is also because they may be ambivalent and hard to measure, and thus are removed from the view of the bigger picture. However, the reality is that livestock are just as intimately connected, at times essential with people’s livelihoods, cultures, and societies as they are with the better-studied health, food, nutrition, environment, and economic sectors. Due to these myriad connections, it is particularly important that potential trade-offs as well as synergies in developing sustainable livestock systems for lower-income societies are analyzed and addressed, both to jump on emerging opportunities and to avoid unintended consequences.

Numerous case studies in almost any socio-cultural context prove that significant improvements of productivity of livestock farming are both possible and desirable within the smallholder context (see textbox for some examples). But it can only be successful if the whole contextual dimensions are considered and are well integrated into business models and value chains.

## Conclusion and Outlook

While the aggregated picture of the size of the economic challenge for sufficient animal-sourced food production may look daunting, the granular conditions on the ground give rise to optimism. Many years of research and improvement in the institutional and policy frameworks are paying off on the ground. Opportunities are created and utilized. The business case studies in the adjacent text box anecdotally illustrate that higher investments in higher productivity livestock systems do not automatically come at the expense of environmental degradation, and moreover, are well capable of supplying bottom of the pyramid customers in low-income countries with cost effective and affordable sources of protein and nutrients. The implementation of a host of technologies increases livestock performance faster than at any time in history. Advances in breeding selection, in feed composition, in digitalization enabled precision livestock farming, and in financial instruments available to the farmer are opening large and lucrative spaces for private investment on any scale, from the smallholder to the venture investor. There are many paths forward which promise success, especially when livestock farming is made to be part of the solution.

## Case Studies Illustrating the Potential for Livestock Around the World to Nourish People and Protect the Environment

Poultry: Ethiochicken is a privately-owned company in Ethiopia which combines a robust dual-purpose poultry breed developed by best-in-class animal genetics technologies, with advanced feed-, vaccination-, and farm management methods. Within only 5 years from 2015 to 2020, the company has tripled the per person egg supply throughout Ethiopia, especially in rural communities. Ethiochicken birds are four to five times more productive than traditional village chicken, and thus make much more efficient use of rural community resources of feed, water, and infrastructure. The company enabled the formation of 8000+ small enterprises and strengthened the socioeconomic livelihood of at least 4 million rural small-scale farmers in the country. Most beneficiaries of these improvements are the livelihoods of women and children as they are the usual livestock keepers in these households. Higher resource efficiency, poverty alleviation, and better nutrition leading to healthier people all combine to reduce the pressure on environmental resources in the rural communities. Ethiochicken kept on generating high business growth also in 2021 and 2022, despite the pandemic and the military conflict in the Northern part of the country.

Dairy: The Embrapa experimental dairy farm in Sao Carlos, Brazil seeks to demonstrate economic and social sustainability. The dairy cows are grazing in two separate areas: during the day in a field with row-planted trees which provide shade and grow biomass, and during the night in an open field with only tropical grass. The cows arrive by themselves twice a day to an automated milking parlor, where a robot performs all milking activity without human intervention. Upon exit the cows will be directed by fencing to another area. The overall system is designed to be climate neutral with carbon sequestration in the soils and in the tree biomass, to maximize animal welfare as the animals roam and graze freely, and to be socially sustainable as only a minimum amount of human labor is required to run the  operation.

Cattle: Shangani in Zimbabwe is a 65,000-ha ranch with no internal fencing situated in natural savannah shrub land, receiving annual rainfall of about 600 mm. Its carrying capacity for commercial cattle is around 16,000 head. The herd is managed in groups of around 200 animals each, which are rotationally grazed on an unmarked paddock system. The animals are in a protected kraal during the night. Amidst the commercial cattle, the ranch also supports a wild group of 300 male elephants who roam the ranch during winter, and about 600 wild giraffes (changes seasonally). There are also several thousand heads of different antelope species, including elands, kudus, nyalas, impalas, and more. A wild zebra population of more than 1,000 head also populates the ranch. The wildlife is not hunted. It is kept in balance by may be 150 wild leopards on the ranch. All typical bushland bird species are prevalent. While the commercial cattle graze the grasses, the wildlife keeps the growth of bush under control, and in this way coexist in ecological synergy. The elephants are kept away from gardens and residential houses on the ranch through trenches that are half a meter wide and one meter deep. As there is no hunting or harassing, wildlife and humans cohabitate safely without aggression but at respectful distance. Measurements have shown that the carbon sequestration of the rotational grazing fully compensates the methane emissions of the cattle.

Pigs: Hamletz is a private breed brand developed by Ms Annechien ten Have. On her farm operations with 600 sows and 5,000 finishers in northern Netherlands she breeds her own type of pigs. The local brand allows her to generate higher prices for her output. The pigs operate as part of a largely circular system, where almost all feed of wheat, sugar beets, and corn are grown on the farm or nearby, and where the animal manure is either recycled on the fields or burned in its 1,1 MW biogas plant. Ms ten Have is the first pig producer in the Netherlands to produce to the standards of the “Better Life Two Stars” requirements, providing the pigs with respective space and living circumstances.

Tilapia aquaculture: Victory Farms built up a 10,000-ton annual production capacity of tilapia fish farming in Lake Victoria in Kenya since 2015. By deploying best-in-class aquacultural technology, and in conjunction with a proprietary retail system of reaching the final consumer, the company produces the most affordable animal-protein food for Kenya’s bottom of the pyramid consumers. Due to its economy of scale, the aquacultural operation can perform to the highest environmental standards in Lake Victoria, and at the same time relieve pressure from overfishing of the lake’s wild fish population. Victory Farms cooperates with not-for-profit Conservation International to foster and report improvements in biodiversity that evolved in their area since operations began. Global tilapia production has doubled over the last ten years, due to the high performance of this fish species to convert low value grains into high value animal proteins.

Sheep: CAFRE hill farm is a 960 ha farm on marginal agricultural land in Ireland. The hill site is exposed to harsh weather influences and includes stretches of bogland. The farm operation makes best use of this land with a breeding ewe flock of 1,100 animals, split roughly in half with Scottish Blackface and crossbred animals composed of Blackface, Swaledale, and Texel. While the farm itself uses minimal amounts of inputs, relying mostly only on the grass habitat of the hills, it uses intensive data science to monitor individual animal performance. All animals are electronically tagged, and their data are tracked in a database for analysis. With the data, the farm optimizes its genetics, the health performance of the animals, and the stocking/paddock systems. CAFRE aims to wean 0.7 kg of lamb for every 1 kg of ewe liveweight, which is the major economic driver of commercial success.

## About the Author(s)

**Isabelle Baltenweck** is the Program Leader, Policies Institutions and Livelihoods at International Livestock Research Institute and Lead of the CGIAR Initiative Sustainable Animal Productivity for Livelihoods, Nutrition, and Gender Inclusion SAPLING. Based in East Africa, she is a development economist with twenty years of post-doctoral experience in agricultural systems in Africa, South and South-East Asia, with a focus on livestock value chains. She is working at the interface of research and development. Her work has a direct relevance to the activities of development practitioners and the private sector, and resulted in publications targeted at that audience, in addition to scientific publications.

**James Blignaut** is a professor extraordinaire attached to the SPL of the Stellenbosch University and an honorary research associate of SAEON. He is also the director of ASSET Research, Restore Africa Fund manager, and is the originator of ReStory. He is a member of the Scientific Council of the World Farmers Organisation. He started his career at the South African Reserve Bank in 1992 as a statistical economist. After joining the University of Pretoria in 1996 he focused his research on the economics of restoration, including the economics of conservation and regenerative agriculture, natural resource accounting and payments for ecosystem goods and services and other financial instruments that can contribute towards society-wide healing.

**Peer Ederer** is the founder and director of GOALSciences, the Global Observatory of Accurate Livestock Sciences, which has the mission to research and communicate scientific evidence about the role of animals in the global food system. He has an MBA degree from Harvard University, a PhD in financial economics and holds an adjunct professorship for innovation studies. He has extensive experience in strategy advisory to private companies and public bodies in the global food system and is a frequent presenter on related topics. He has been engaged in scientific research in cooperation with globally leading universities from around the world and is a member of the Scientific Council of the World Farmers Organisation.

**Celso Moretti** is President of Embrapa, the Brazilian Agricultural Research Corporation. He has a BSC in agronomy from Universidade Federal de Viçosa, a master’s degree in plant science from Universidade Federal de Viçosa (1993), a PhD in plant science from Universidade Federal de Viçosa (1998), and is alumni of the Harvard Kennedy School of Government of Harvard University. He has also been a Courtesy Associate Professor with the University of Florida, the USA, since 2006. He has extensive research and experience in the science and technology of food production, focusing on after harvest physiology. In 2021, he received the Norman Borlaug Award in the category “Sustainability”.

**Shirley Tarawali** is Assistant Director General at International Livestock Research Institute (ILRI, Kenya) a CGIAR centre and is Chair of the Global Agenda for Sustainable Livestock. Shirley’s portfolio includes representation, and engagement within ILRI and beyond to support sustainable livestock contributions to the wider development agenda. She coordinated ILRI’s strategy process and led the inception and growth of ILRI’s portfolio on sustainable livestock advocacy and communications. Shirley has over 35 years of experience implementing and leading livestock research for development, has authored or contributed to over 100 publications and holds a PhD in Plant Science from the University of London.
